# Enhancing Insight into Photochemical Weathering of Flax and Miscanthus: Exploring Diverse Chemical Compositions and Composite Materials

**DOI:** 10.3390/molecules29163945

**Published:** 2024-08-21

**Authors:** Roland El Hage, Raíssa Carvalho Martins, Clément Brendlé, Dominique Lafon-Pham, Rodolphe Sonnier

**Affiliations:** 1 PCH, IMT Mines Alès, 6 Avenue de Clavières, 30100 Alès, France; raissacarvalho1@gmail.com (R.C.M.); clement.brendle@mines-ales.fr (C.B.); rodolphe.sonnier@mines-ales.fr (R.S.); 2 EuroMov Digital Health in Motion, University Montpellier, IMT Mines Ales, 6 Avenue de Clavières, CEDEX, 30319 Alès, France; dominique.lafon@mines-ales.fr

**Keywords:** lignocellulosic biomass, chemical composition, PP composites, accelerated weathering, color monitoring, thermal stability

## Abstract

The accelerated weathering of flax and miscanthus fibers possessing distinct chemical compositions was investigated. The chosen fibers included raw, extractive-free (EF) and delignified samples (x3), alone and used as fillers in a stabilized polypropylene blue matrix (PP). Modifications in both color and the chemical composition of the fibers throughout the weathering process under ultraviolet (UV) light were meticulously tracked and analyzed by spectrophotometry and attenuated total reflectance with Fourier-transform infrared spectroscopy (ATR-FTIR). The inherent nature and composition of the selected fibers led to varied color-change tendencies. Raw and EF flax fibers exhibited lightening effects, while raw and EF miscanthus fibers demonstrated darkening effects. Extractives exhibited negligible influence on the color alteration of both flax and miscanthus fibers. This disparity between the fibers correlates with their respective lignin content and type, and the significant formation of carbonyl (C=O) groups in miscanthus. Better stability was noted for delignified flax fibers. A comparative study was achieved by weathering the PP matrix containing these various fibers. Contrary to the weathering observations on individual fibers, it was noted that composites containing raw and EF flax fibers exhibited significant color degradation. The other fiber-containing formulations showed enhanced color stability when compared to the pure PP matrix. The study highlights that the UV stability of composites depends on their thermal history. As confirmed by thermogravimetric analysis (TGA), fiber degradation during extrusion may affect UV stability, a factor that is not apparent when fibers alone are subjected to UV aging.

## 1. Introduction

In recent years, the automotive industry has increasingly prioritized vehicle weight reduction, sustainability and ecological awareness [1]. To address these concerns, there has been a growing emphasis on integrating renewable natural resources, particularly plant-based fibers, in the development of lightweight and eco-friendly materials [2]. 

However, one inherent challenge lies in the fact that native lignocellulosic fibers and polymeric matrices undergo alterations when used in outdoor applications, being influenced by natural weathering and climatic conditions [3]. The components of lignocellulosic resources are susceptible to photodegradation. These materials consist of various constituents, including extractives such as fatty acids, resins, waxes, gums, terpenes and pectins, as well as polysaccharides like cellulose and hemicellulose, and polyphenols such as lignin. Among these components, extractives play a significant role in inducing color changes in lignocellulosic biomass, such as wood. These moieties are susceptible to light due to their composition, which is based on polyphenols and terpenoids, among others [4,5,6,7]. Lignin molecules are also susceptible to photodegradation. Since 1945, several researchers have attributed the yellowing and discoloration of pulps to photodegradation and the reactivity of lignin [8,9,10,11,12]. Hemicellulose, especially arabinoglucuronoxylan, could also be impacted by UV radiation [13]. Cellulose and most of its derivatives are also light-sensitive, even if they are quite transparent in the visible and near ultraviolet regions [14,15,16]. 

Among various thermoplastic matrices, polypropylene (PP) is widely favored due to its resilience against chemical and biological degradation. Despite lacking inherent chromophores, this polyolefin is susceptible to photodegradation [17,18]. The photo-oxidation process of polypropylene (PP) primarily commences with the formation of hydroperoxide groups, which subsequently undergo photolysis, resulting in the creation of carbonyl, esters and hydroxyl chromophoric groups [18,19]. Additionally, the presence of residual heterogeneous organo-aluminum–titanium and metallic oxide catalysts catalyzes the decomposition of hydroperoxides [11]. Active oxygen compounds like atmospheric ozone (O_3_) and aromatic impurities absorbed from the environment may also act as potential photoinitiators in PP degradation [11]. During aging, two competitive mechanisms, namely chain scission and cross-linking, are observed. The main modifications of PP include weight change, loss of transparency and gloss, discoloration and mechanical breakdown. To mitigate this aging phenomenon, ultraviolet light absorbers and antioxidants are commonly employed [20,21]. 

For fiber-reinforced composites, a multitude of factors such as fiber content, coupling agents, additives, stabilizers, manufacturing methods, and of course weathering conditions can significantly influence photodegradation [3,22,23,24]. Notably, injected molded samples tend to exhibit slower degradation due to the presence of a polymer-rich surface layer [25]. The phenomenon of color fading under wet artificial weathering conditions is often associated with a leaching effect, wherein degraded extractives/lignin and other water-soluble products are washed away [26]. Peng et al. (2015) [27] confirmed an increase in the surface roughness of PP composites reinforced with wood flour, lignin and cellulose using atomic force spectroscopy (AFM) analysis. Furthermore, the crystallinity of PP, in both pure and reinforced samples, tends to increase after weathering, attributed to polymer chain scissions and recrystallization. Notably, prolonged weathering time induces significant surface chemistry changes due to the photodegradation and photo-oxidation of lignin and cellulose, as highlighted in this study. 

Moreover, various lignocellulosic resources, including wood fibers, cellulose, wheat straw, hemp, palm fibers and kenaf, among others [3,23,24,27,28], have been combined with pure PP matrices to produce reinforced PP composites. Investigations into the weathering of PP composites reinforced with short or unidirectional flax fibers have revealed significant effects on the surfaces of these composites and color change [24,29,30]. However, research on the photodegradation of PP miscanthus composites remains limited. Ragoubi et al., 2012 [31] explored the mechanical and thermal properties of composites based on miscanthus fibers and a polypropylene matrix. Utilizing the corona technique, a physical surface modification treatment based on a high-voltage electrical discharge, enhanced the interfacial compatibility between the matrix and the fillers. Girones et al., 2016 [32] demonstrated the promising use of stem miscanthus in PP composites, highlighting the effective reinforcement of the PP matrix combined with PP-g-MA without chemical modification of the miscanthus stem. Miscanthus fragments with the required surface area can be obtained through a simple milling process to achieve a better interphase. Despite these advancements, there remains a dearth of information regarding the durability of miscanthus fibers and their composites with varying chemical compositions under artificial weathering conditions.

This study aims to explore the accelerated artificial weathering of flax and *Miscanthus x giganteus*, a sterile hybrid of *Miscanthus sinensis* and *Miscanthus sacchariflorus* (a C4-type crop) known for its non-invasive nature and minimal requirements for herbicides and nitrogen. The investigation compares the weathering behavior of *Miscanthus x giganteus* stem fragments with short flax fibers, both before and after their integration into a stabilized PP-based blue matrix. The decision to use a PP-based blue matrix instead of biobased poly(ethylene terephthalate) (PET) [33], for example, was primarily driven by our industrial partner’s requirements and constraints. The selection of a stabilized colored matrix was made to closely resemble materials with commercially viable compositions for automotive applications, such as car bumpers or other external parts [34]. This study specifically focuses on assessing the color variation in samples rather than delving into their mechanical properties. Both the individual fibers and the composites underwent weathering from one side, which was exposed to UV radiation. The roles of extractives and lignin are emphasized, prompting the investigation into the impact of chemical composition on fiber color modification. To this end, compressed discs of raw fibers (Raw), extractive-free fibers (EF) and delignified fibers (x3) underwent artificial aging to discern the influence of chemical composition on fiber color alteration. Additionally, these fibers were incorporated into a PP-based matrix and the resulting composites underwent aging to gauge the fibers’ effect on composite color change. Throughout this study, color variation was monitored using a spectrophotometer based on the CIE L*a*b* system. Complementary techniques such as ATR-FTIR, scanning electron microscopy (SEM) and thermogravimetric analysis (TGA) were employed to substantiate our observations regarding color change.

## 2. Results

### 2.1. Fiber Analysis 

Table 1 presents the elemental analysis of C, H, N and S in raw flax and miscanthus. The results indicate the absence of nitrogen and sulfur elements, along with a comparable low hydrogen content for both fibers. However, there is a higher carbon content in miscanthus fibers, suggesting a higher presence of lignin [35]. 

**Figure 1:**
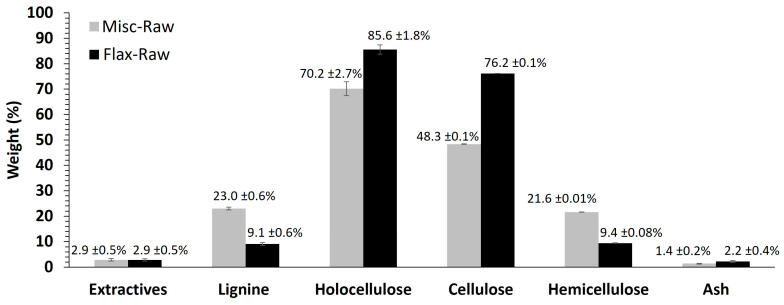
Chemical composition (wt %) of flax and miscanthus fibers.

Various miscanthus and flax fibers with diverse chemical compositions were prepared to assess their impact on the weathering process, both individually and within composite materials. The chemical compositions of Flax-Raw and Misc-Raw, determined through various weight characterization methods (as detailed in Section 3.2.2, Section 3.2.3, Section 3.2.4, Section 3.2.5 and Section 3.2.6), are depicted in Figure 1.

**Figure 2:**
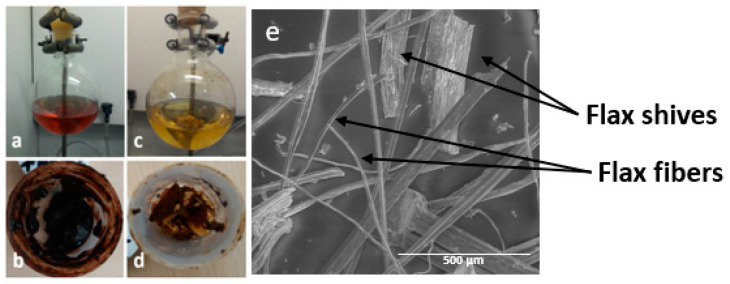
Photographs of (**a**,**c**) extractives of miscanthus and flax, respectively, in the solvent, and (**b**,**d**) extractives of miscanthus and flax, respectively, after solvent removal. (**e**) Micrograph of native flax obtained by SEM observations.

The extractive and ash content are similar for both fibers. However, a notable distinction arises concerning the color and texture of the extractives, as depicted in Figure 2a–d. Miscanthus extractives manifest as red in a soluble state and as viscous dark red in a solvent-free state (Figure 2a,b), while flax extractives appear yellow in a soluble state and as a friable solid with a yellow core and a darkened surface after solvent evaporation (Figure 2c,d). Miscanthus extractives comprise saturated, unsaturated and *α*-hydroxy fatty acids, along with aromatic compounds such as vanillin, syringaldehyde, *p*-coumaric acid, ferulic acid and *p*-hydroxybenzaldehyde, among others [36]. On the other hand, flax extractives encompass *n*-alkanes, fatty alcohols, *n*-aldehydes, fatty acids, *α*-hydroxy fatty acids, free sterols/triterpenols, sterol/triterpenol esters, sterol glycosides, steroidal hydrocarbons, steroid ketones/triterpenoids and waxes of esters [37,38]. The red color of miscanthus extractives predominantly arises from the presence of anthocyanin flavonoids, notably 3-deoxyanthocyanins [39]. Conversely, the yellow hue of flax extractives is primarily attributed to the main fatty and waxy compounds identified, along with other flavonoid derivatives of the C-glucoside type, such as vitexin, orientin and isoorientin [38,40,41]. It is noteworthy that miscanthus exhibits a significantly higher lignin content (23.0 ± 0.54%) compared to flax (9.1 ± 0.58%), a finding consistent with the results obtained from elemental analysis. Consequently, the holocellulose content, representing the polysaccharide portion comprising cellulose and hemicelluloses, is higher in flax (85.6 ± 1.8%) compared to miscanthus (70.2 ± 2.7%). A notable discrepancy is observed in the polysaccharide composition, where the hemicellulose content is higher in miscanthus (21.6 ± 0.01%) compared to flax (9.4 ± 0.08%). While the percentages of the various constituents of the two fibers align with the existing literature, there appears to be an exception regarding the lignin content of flax fibers, which appears higher in our study [42,43,44]. This elevated lignin content is attributed to the presence of flax shives, as confirmed by SEM observations (Figure 2e), which possess higher lignin content than the flax fibers themselves [45].

After treatment, there are notable changes in the chemical composition of the fibers. Table 2 presents the Klason lignin content of the various fibers. The removal of extractives results in a slight increase in lignin content, by 2% and 4% compared to miscanthus and native flax, respectively. Additionally, the application of the bleaching treatment (x3) leads to the elimination of 90% of the initial lignin content in both flax and miscanthus fibers.

**Figure 3:**
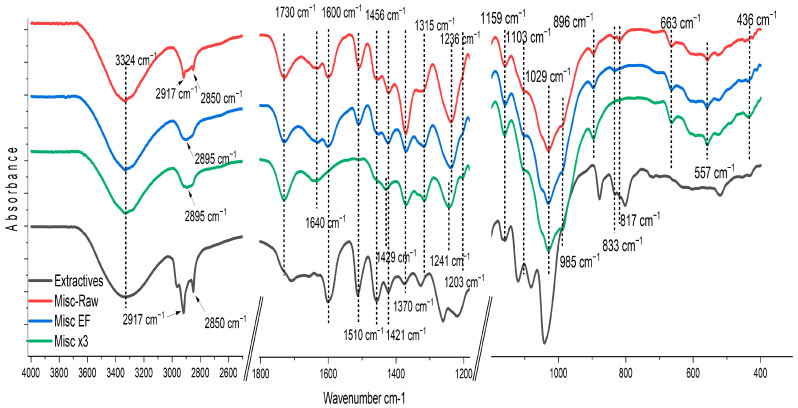
FTIR spectra of Misc-Raw, Misc EF, Misc x3 and extractives.

The various functional groups present in the fibers were analyzed using FTIR spectroscopy (Figure 3). Detailed FTIR signals and their corresponding assignments are provided in the Supporting Information S1. It is noteworthy that no clear modification was observed by FTIR for raw or treated flax fibers. This observation could be attributed to flax’s lower lignin content or its nature, which primarily drive the changes observed in the spectra. Therefore, the analysis will be primarily focused on the miscanthus spectra. 

In the Misc-Raw spectrum (Figure 3—red curve), the broad band at 3324 cm^−1^ corresponds to the hydroxyl groups (O-H) present in cellulose and hemicellulose, with signals from lignin hydroxyl groups (-OH) also contributing to this band [46,47]. Extractives are also involved in this zone, as shown by the strong band at 3324 cm^−1^ (Figure 3—black curve) assigned as a hydroxyl group (O-H) in the phenolic and aliphatic structures. 

The bands at 2917 cm^−1^ and 2850 cm^−1^ observed in the raw miscanthus spectrum correspond to the asymmetric stretching vibration of -CH_2_ and the symmetrical stretching vibration of aliphatic CH_3_ present in the waxy extractives. The absorption band at 2985 cm^−1^ for the three samples is assigned to the C-H or CH_2_ stretching of cellulose and hemicellulose [47]. The C-H stretching of lignin could also be involved in this band [46]. The band at 1730 cm^−1^, corresponding to the carbonyl groups (C=O stretching), is present in the three samples and could be attributed to acetyl groups or to the carboxylic esters (acyl groups) of hemicellulose. The broad band observed at 1640 cm^−1^ could be related to C=O stretching carbonyl and the ethylene p-coumaryl ester group in lignin [42]. However, H-O-H deformation from cellulose-and-hemicellulose-adsorbed water seems to also be involved in this zone [48,49]. C=C stretching vibrations and C-H deformation from aromatics (lignin) are detected at 1600 cm^−1^/510 cm^−1^ and 1456 cm^−1^, respectively. In the fingerprint zone (<1500 cm^−1^), the absorbance bands at 1421 cm^−1^ and 1370 cm^−1^ could be assigned to the H-C-H/O-C-H bending vibration in cellulose. These bands could also be associated with aliphatic C-H stretching in methyl and phenol OH, and with aromatic skeleton deformation caused by the presence of lignin and extractive moieties [46,50]. The band at 1315 cm^−1^ might be attributed to the CH_2_ rocking vibration in cellulose. The band at 1240 cm^−1^ could be attributed to C-O stretching from lignin (guaiacyl ring), cellulose and hemicellulose. The band at 1159 cm^−1^ corresponds to the C-O-C asymmetrical stretching in cellulose and hemicellulose. This band could also be impacted by the presence of extractives, as shown in the obtained IR spectrum of pure extractives (Figure 3—black curve), in addition to a weak contribution of the C-H band in miscanthus lignin at 1166 cm^−1^ [46,51]. The bands at 1103 cm^−1^, 1029 cm^−1^ and 985 cm^−1^ correspond to the C-C, C-OH and C-H ring and side group vibration in cellulose and hemicellulose. The band at 989 cm^−1^ is assigned to the C-O-C, C-C-O and C-C-H deformation and stretching in cellulose. The small band at 833 cm^−1^ present in the raw and extractive-free samples is assigned to the C-H band’s out-of-plan deformation of the guaiacyl group in lignin [46,51]. The bands at 663 and 558 cm^−1^ are attributed to C-OH’s out-of-plan bending in cellulose [52]. 

The spectrum of Misc EF (Figure 3—blue curve) exhibits the same features as Misc-Raw, except for the absence of bands at 2917 cm^−1^ and 2850 cm^−1^ due to the removal of waxy extractives. It is worth noting that these bands could also be observed in the FTIR spectra of miscanthus lignin, as noted by Bergs et al. in 2019 [46]. However, in this study, extractive-free samples did not exhibit these bands, despite the presence of lignin. The spectrum of Misc x3 (Figure 3—green curve) exhibits the absence of bands at 2917 cm^−1^ and 2850 cm^−1^ due to wax elimination, along with the disappearance of some bands attributed to lignin extraction. Notably, the band at 1730 cm^−1^ (C=O stretching of carbonyl groups) remains clearly visible with no alteration in the band shape despite the elimination by the delignification of the carbonyl groups in miscanthus lignin typically observed at 1705–1708 cm^−1^ [42,46], as well as the acetate carbonyl group at 1739 cm^−1^ [42,47]. This suggests that the band at 1730 cm^−1^ is primarily associated with the carbohydrate component. The disappearance of the bands at 1600 cm^−1^, 1510 cm^−1^ and 1456 cm^−1^, which are linked to aromatics (C=C stretching vibrations and C-H deformation), following delignification, indicates their association with aromatic lignin. Additionally, it could be inferred that these bands are partly influenced by extractives in raw fibers containing minimal amounts of polyphenols [53]. The disappearance of these bands in the delignified sample highlights the absence of extractives and lignin, as noted by Salim et al., 2021. The band observed at 1421 cm^−1^ shifts to 1429 cm^−1^ for bleached samples and is primarily attributed to the H-C-H bending vibration in cellulose following lignin extraction. Referred to as the “crystallinity band”, this shift may indicate modifications in cellulose crystallinity after lignin extraction, as highlighted by Salim and coworkers [54]. This alteration could suggest the absence of lignin and the transformation of amorphous cellulose and crystallized cellulose type II at 1421 cm^−1^ to crystalline cellulose type I at 1429 cm^−1^, as proposed by Liu and coworkers [55].

It is noteworthy that the band at 1370 cm^−1^ remains nearly unchanged after lignin extraction (Misc x3) in comparison to the Misc EF sample, despite being traditionally associated in the literature with the aromatic skeleton of miscanthus lignin [46]. This observation underscores the contribution of carbohydrates to the absorption in this region. Additionally, the band at 1315 cm^−1^ (CH_2_ rocking vibration in cellulose [47]) appears sharper after lignin extraction. This alteration may be attributed to the removal of aromatic stretching and C-O groups from lignin, which typically manifest around 1331 cm^−1^ [46]. Finally, it is notable that the disappearance of the band at 833 cm^−1^, assigned to the C-H out-of-plane deformation of the guaiacyl group in lignin [46,51], further underscores the effective removal of lignin.

### 2.2. Compressed Fiber Disc Weathering 

**Figure 4:**
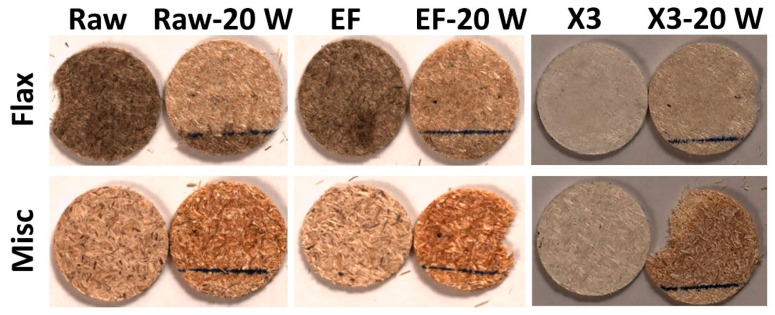
Photographs of miscanthus (Misc) and flax fibers (raw, EF, x3) before and after 20 weeks of weathering. (The line drawn on the samples marks the area that was partially shielded from UV exposure due to the way the samples were mounted in the holder. The area below the line was excluded from the analyzed surface area, as it was not exposed.)

Flax and miscanthus compressed discs underwent UV weathering for up to 20 weeks, revealing distinct color changes, as depicted in Figure 4. Flax and miscanthus exhibit divergent responses to weathering-induced color variation. While the surface color of raw and EF flax lightened and discolored, raw and EF miscanthus developed a brownish hue after 20 weeks of weathering. Furthermore, delignified flax (Flax x3) exhibits a fading white color, while delignified miscanthus (Misc x3) demonstrates a darkening hue. This contrasting color behavior between the fibers is attributed to their distinct chemical compositions, as suggested by the existing literature. The observed discoloration in flax (Raw and EF) aligns with findings for jute and sisal fibers [56], indicating a potential association with the intensified hydrolysis of lignin through photo-oxidation. In contrast, the miscanthus samples (Raw, EF and x3) exhibit a browning effect, possibly associated with lignin modification and the generation of new carbonyl groups, such as ketones, aldehydes and quinones [57]. This hypothesis will be further explored through a detailed analysis of the FTIR spectra. The pronounced color alteration observed in miscanthus x3 may be attributed to its higher residual lignin content (2.5 wt%) compared to flax x3 (0.85 wt%).

**Figure 5:**
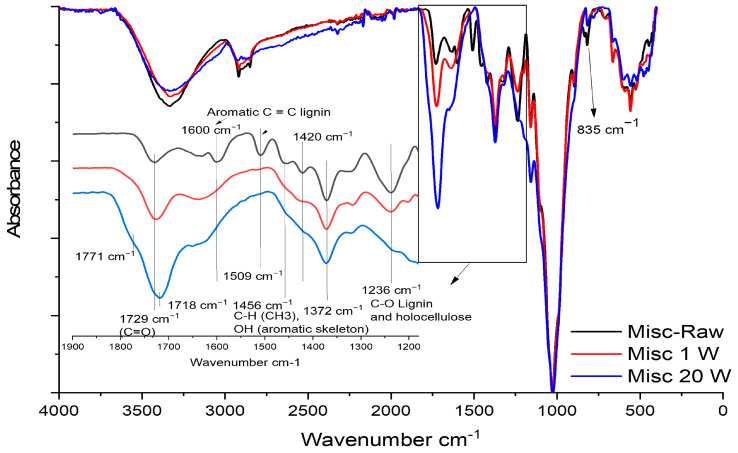
FTIR spectra of Misc-Raw before and after 1 week and 20 weeks of weathering.

For a better understanding of our results, the FTIR spectra of raw miscanthus before and after weathering were analyzed (Figure 5). Note that the spectra of Misc EF shows same tendency as that of Misc-Raw. The changes in the FTIR spectra of Flax-Raw, Flax EF and Misc x3 (Supporting Information S2–S5) were also similar before and after weathering, probably because most of the changes were related to lignin, of which these fibers contain a low content. Figure 5 presents the spectra of raw miscanthus before and after 1 week and 20 weeks of weathering. It is notable that the bands at 2917 cm^−1^ and 2850 cm^−1^, which are attributed to the vibration of -CH_2_ and -CH_3_ present in the waxy extractives, appear to decrease significantly after 20 weeks of prolonged weathering. This indicates the degradation of extractives over time.

The most significant variation in the FTIR spectra was observed in the wavenumber range of 1900–1200 cm^−1^. Figure 5 provides a closer look at this zone. The band at 1730 cm^−1^, corresponding to carbonyl groups (C=O), exhibits a slight shift to 1718 cm^−1^ after 20 weeks of UV weathering, along with a significant increase in intensity. Additionally, a new shoulder band emerges at 1771 cm^−1^. This emergence could be attributed to the formation of new carbonyl groups resulting from lignin photo-cleavage and the transformation into o- and p-quinonoid containing unsaturated carbonyl compounds [16,58,59]. This observation is accompanied by the disappearance of bands at 1600 cm^−1^, 1510 cm^−1^ and 1456 cm^−1^, which underscores the degradation of the aromatic skeleton of lignin starting from the first week of weathering. Moreover, the band at 1240 cm^−1^, assigned to the asymmetric stretching of ether bonds (C-O), progressively decreases with time and becomes barely noticeable after 20 weeks. This suggests the degradation of both lignin and polysaccharides. The band at 1421 cm^−1^ shifts slightly to 1429 cm^−1^, accompanied by a progressive decrease in intensity. This phenomenon, observed previously after the delignification of raw miscanthus, indicates lignin degradation and the modification of cellulose crystallinity [55]. The band at 1370 cm^−1^ remains unchanged during weathering. The band at 835 cm^−1^, present in native miscanthus, disappears from the first week of weathering, indicating once again the degradation of guaiacyl units in lignin [46,51].

**Figure 6:**
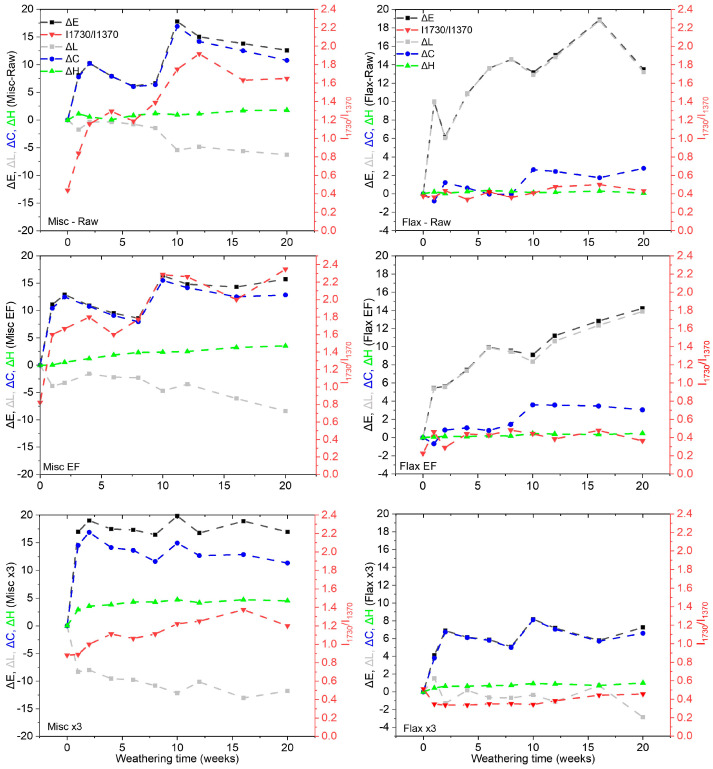
Color variation ($∆E_{ab}^{*}$), lightness (∆$L^{*}$), chroma (∆$C_{ab}^{*}$), hue (∆$H_{ab}^{*}$) and carbonyl ratios (I_1730_/I_1370_) of flax and miscanthus (Raw, EF and x3) as a function of weathering time (weeks) (curves are added as a guideline to easily follow the results’ evolution).

The variations in color ($∆E_{ab}^{*}$), lightness (${∆L}^{*}$), chroma ($∆C_{ab}^{*}$) and hue ($∆H_{ab}^{*}$) of the miscanthus and flax fibers (Raw, EF and x3) are presented in Figure 6.

The total color variation ($∆E_{ab}^{*}$—depicted in Figure 6 with black curves) reflects the measured change in fiber color before and after weathering at specific intervals. In all cases, $∆E_{ab}^{*}\;$ exceeded the threshold value of 2, known as the “limit for the human eye to recognize a color change,” and the threshold value of 5, which is the “limit value for color to be clearly noticed by the human eye as two different colors” [60]. Across all fibers examined, changes became visibly apparent as early as the end of the first week of the aging process. Notably, Flax x3 showed the least visible change. Interestingly, the color change for all fibers did not progress monotonously. For miscanthus fibers, regardless of treatment, a local minimum was observed between 5 and 10 weeks of aging. It should be noted that the modification of $∆E_{ab}^{*}$ values is not always attributable to the same component. Depending on the type of fibers, $∆E_{ab}^{*}$ is influenced by changes in lightness (${∆L}^{*}$), chroma ($∆C_{ab}^{\;*}$) and hue ($∆H_{ab}^{*}$) components, which vary differently. In other words, for instance, the $∆E_{ab}^{*}$ of different samples could exhibit similar values, which might be associated with an increase or decrease in ${∆L}^{*}$ in one case and an increase or decrease in $∆C_{ab}^{\;*}$ in another case. Therefore, the variation in $∆E_{ab}^{*}$ will be initially discussed separately, followed by a discussion concerning the ${∆L}^{*}$, $∆C_{ab}^{\;*}$ and $∆H_{ab}^{*}\;$ components to provide a better understanding of the origin of variation in $∆E_{ab}^{*}$ values.

Upon comparing the evolution of flax and miscanthus with and without extractives (raw and EF), it appears that $∆E_{ab}^{*}$ increases for both fibers. After 20 weeks of weathering, $∆E_{ab}^{*}$ for Misc-Raw and Misc EF reached values of 13 and 15, respectively. Similarly, $∆E_{ab}^{*}\;$ for Flax-Raw and Flax EF reached values of 13 and 14, respectively, for the same aging period. Interestingly, the values remain very close with and without extractives. Therefore, the absence of extractives seems to have a limited impact on $∆E_{ab\;}^{*}$ values during weathering. Indeed, the situation differs for other lignocellulosic biomasses. In wood, color variation is often attributed to structural changes in the molecules present in extractives or to the recombination of phenolic compounds [4]. For Scots pine wood, the photodegradation of thermally modified wood during weathering was influenced by the presence of extractives, and wood containing higher extractive levels experienced more severe color changes during weathering [5]. The alteration in color was linked to changes in the structure of extractives, including a reduction in terpene concentration during wood aging [7]. Consequently, photodiscoloration involves intricate mechanisms that may entail structural alterations in the functional groups of wood extractive molecules [4,61]. It is notable from the current study that the extractives present in flax and miscanthus fibers may not be the primary components responsible for color variation.

The behavior of $∆E_{ab}^{*}$ for delignified fibers (Misc x3 and Flax x3) exhibits distinct patterns. Particularly, the $∆E_{ab}^{*}$ value of Misc x3 experiences an abrupt variation from the first week of weathering, stabilizing around a plateau near the value of 17 up to 20 weeks. In contrast, Flax x3 demonstrates a gradual increase in value from the initial week of weathering ($∆E_{ab}^{*}$ = 4) and reaches a value of 7 after 20 weeks. This difference in behavior suggests better color stability for delignified flax (flax x3) compared to delignified miscanthus, possibly attributable to the higher residual lignin content in Misc x3 (2.5 wt%) compared to Flax x3 (0.85 wt%).

To delve deeper into the understanding of the $∆E_{ab}^{*}$ variation, factors such as hue ($∆H_{ab}^{*}$), lightness (${∆L}^{*}$) and chroma ($∆C_{ab}^{\;*}$) are delineated in Figure 6. Hue ($∆H_{ab}^{*}$—depicted by the green curve) demonstrates a minimal increase for miscanthus (raw, EF and x3) and remains relatively unchanged for flax fibers. This observation suggests that hue is not a significant contributor to the color change observed in both fiber types after weathering. The parameters $∆C_{ab}^{\;*}$ and ${∆L}^{*}$ appear to play a more prominent role in the variation of $∆E_{ab}^{*}$, depending on the fiber type. For miscanthus (raw, EF and x3), ${∆L}^{*}$ exhibits a decrease over time (from 0 to −6 for Misc-Raw, from 0 to −8 for Misc EF and from 0 to −11 for Misc x3 after 20 weeks). Conversely, $∆C_{ab}^{\;*}$ experiences a significant increase (from 0 to 13 for Misc-Raw, Misc EF and Misc x3 after 20 weeks), emerging as the primary driver for the observed rise in $∆E_{ab}^{*}$. This trend of $∆C_{ab}^{\;*}$ is indicative of surface color browning or darkening, as observed in Figure 4. 

**Figure 7:**
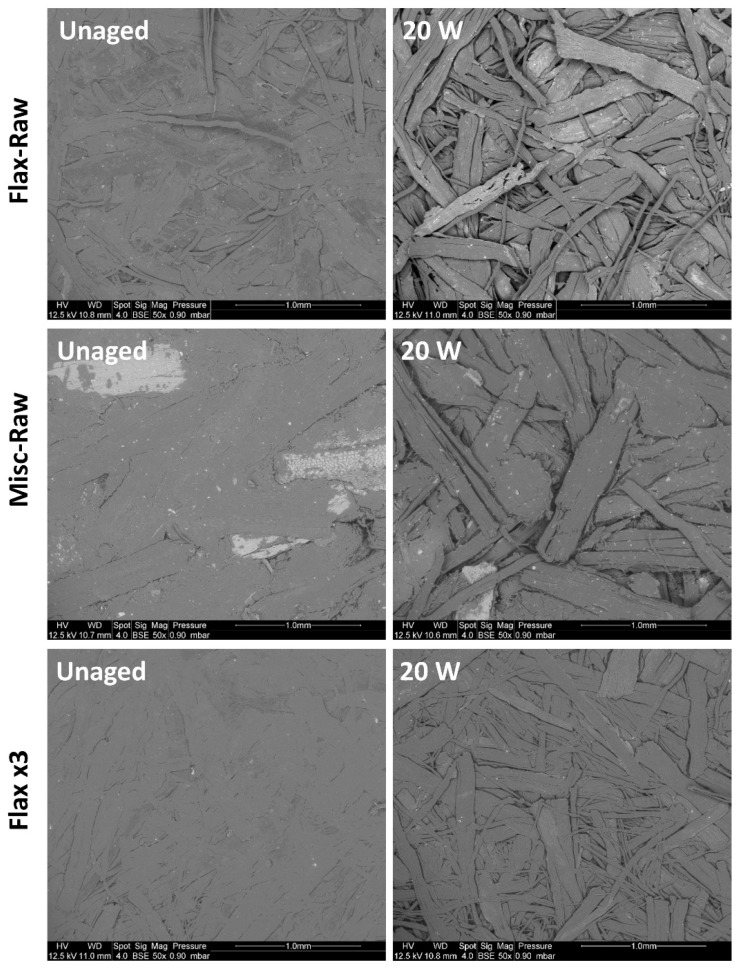
SEM images of Flax-Raw, Misc-Raw and Flax x3 before and after 20 weeks of weathering.

However, for raw and EF flax, the evolution of $∆E_{ab}^{*}$ is primarily driven by an increase in ΔL after 20 weeks (from 0 to 14 for raw and EF after 20 weeks), in addition to the slight increase observed for $∆C_{ab}^{\;*}$ (from 0 to 3 for raw and EF after 20 weeks). This behavior suggests a lightening effect, as observed in Figure 4. The relatively lower increase in $∆E_{ab}^{*}$ for Flax x3 with increasing weathering time is attributed to an increase in $∆C_{ab}^{\;*}$ (from 0 to 6) and a decrease in Δ*L* (from 0 to −3), which is completely different from the trend observed previously for raw and EF fibers. This change in the dependency of $∆E_{ab}^{*}$ components is primarily due to the fiber treatment, which resulted in color lightening induced by the delignification process (residual lignin is only 0.8%) and subsequent color changes after weathering (increase in $∆E_{ab}^{*}\;$ and decrease in ${∆L}^{*})$ in comparison to the raw and EF flax. Therefore, the initial color of the fibers also appears to influence the $∆E_{ab}^{*}$ values after weathering. Moreover, SEM observations of Flax- and Misc-Raw discs (Figure 7) reveal the defibrillation and decohesion of fiber discs, the emergence of porosities due to chip decompression and the elimination of degradation products along with fiber cracking, all of which could have a slight impact on color perception. These observations were less pronounced for Flax x3, indicating a lesser elimination of degradation products and better stability.

For a deeper understanding, considering that the reference bands for carbohydrates at 1370 cm^−1^ remained relatively unaffected by weathering, carbonyl ratios (I_1730_/I_1370_) were computed (Figure 6—red curves) and juxtaposed with color modifications [16,62] for a comparative analysis. Generally, the carbonyl ratios exhibited an upward trend for miscanthus fibers (rising from 0.4 to 1.5/2.3 after 20 weeks), regardless of their composition. This escalation in carbonyl content appears closely tied to the concurrent rise in $∆E_{ab}^{*}$, which itself correlates with $∆C_{ab}^{\;*}$. Conversely, for flax fibers, the carbonyl index appears to be less susceptible to change during and after weathering. Consequently, ${∆L}^{*}\;$ emerges as the primary determinant of $∆E_{ab}^{*}$ evolution for flax.

Therefore, in the case of Misc-Raw and EF fibers, if $∆C_{ab}^{\;*}$ drives the increase in $∆E_{ab}^{*}$, it may be correlated with carbonyl formation. Based on the literature, the most common color modification mechanism is thought to be associated with the oxidation of phenolic species (e.g., aromatic ketones) and the formation of conjugated quinone methide, the elimination of formaldehyde, and the generation of phenoxy radicals in lignin due to their ability to absorb radiation [6,7]. However, if $\;{∆L}^{*}$ is responsible for the increase in $∆E_{ab}^{*}$, carbonyl formation may not be involved. 

The variation in $∆C_{ab}^{\;*}$ observed in miscanthus appears to be associated with its higher lignin content (23 wt% for misc-Raw and EF and 2.5% for misc x3), which acts as a chromophore group upon its conversion into quinoids and the formation of carbonyl compounds. In the case of delignified fibers, aside from the transformation of residual lignin, the initial color of the fibers (white due to delignification) that browns after weathering is also involved in the increase in $∆C_{ab}^{\;*}$. A hypothesis for the variation in ${∆L}^{*}$ of flax (Raw and EF) could be associated with the further photodegradation of lignin by-products [56]. The absence of aromatic lignin bonds in the raw and EF flax in the FTIR spectra, as observed before weathering, could indicate an initial transformation into quinoids due to the retting process before aging. Another explanation could be linked to the oxidation and reduction of lignin into hydroquinone, which could lead to a photobleaching phenomenon and an increase in ${∆L}^{*}\;$ [22]. A third hypothesis could be related to the structure of lignin. Lignin primary precursors consist of phenol units such as p-coumaryl, coniferyl and sinapyl alcohols, which form the basis of lignin’s composition (p-hydroxyphenyl (H), guaiacyl (G) and syringyl (S) units). Miscanthus lignin, as reported by El Hage and coworkers [51], appears to have higher S units (44%), but lower H (4%) and G (52%) units compared to flax fibers (13% H, 72% G, 15% S) and flax shives (5% H, 87% G, 8% S) [63]. This variation in lignin structure may influence the observed color change. 

Furthermore, the browning effect observed in miscanthus could be linked to the photocleavage reaction, which assumes a significant role in lignin photodegradation [11]. Research on the photodegradation of non-phenolic *α*-carbonyl *β*-O-4 lignin model compounds, such as 3,4-dimethoxy-*α*-(2’-methoxyphenoxy)acetophenone), has revealed that *β*-C-O bond cleavage serves as the primary photochemical step, resulting in the formation of phenacyl and guaiacoxy radicals. These cleavage reactions are pertinent to *β*-1 structures, which appear to contribute to coloration through the production of colored oligomeric materials, as observed in Misc (Raw, EF and x3). Thus, it can be inferred that chromophoric products arise from lignin scission. Lignin degradation is believed to occur when *α*-carbonyl absorbs radiation energy and enters an excited state, initiating the cleavage of the *β*-arylether linkage and a series of electron migration steps to form quinoid compounds [12].

The observed color variation in miscanthus could be also attributed to hemicellulose and cellulose photolysis, as evidenced by the alterations in the ether (C-O) band at 1236 cm^−1^ (Figure 5). In the aging process of fir wood, distinct changes in individual hemicelluloses were observed. Research indicated that arabinoglucuronoxylan exhibited less resistance to aging compared to galactoglucomannan. There was a relative decrease in arabinose and xylose, whereas the amounts of galactose, glucose and mannose increased. This suggests that arabinoglucuronoxylan undergoes more rapid degradation compared to galactoglucomannan [13]. Moreover, cellulose photolysis has been extensively studied since 1954 [58], revealing that it induces the rupture of carbon–carbon or carbon–oxygen bonds, consuming energy in the range of 80 to 90 kcal per mole. Research has shown that cellulose irradiation with 2537 A light leads to significant degradation, both in the presence and absence of oxygen. The formation of carboxyl and reducing groups (aldehyde formation) was observed during cellulose irradiation in air, along with the detection of gaseous products such as hydrogen, carbon monoxide and carbon dioxide [64]. These modifications were linked to a decrease in glycosidic linkages, while hydrogen production was associated with the photolysis of alcohol groups. Another significant phenomenon associated with cellulose photolysis, known as photosensitization, involves sensitizers, oxygen and moisture factors. Even if cellulose itself does not absorb light to a significant extent, the presence of impurities capable of light absorption within its structure can induce chemical changes. Based on the work of Egerton and coworkers [15], temperature emerges as a crucial factor impacting the mechanism of cellulose photolysis, particularly in dry oxygen and dry nitrogen atmospheres. Furthermore, relative humidity influences different reaction types in oxygen-containing atmospheres. Short exposure times revealed that cellulose degradation was more pronounced in dry air compared to a wet atmosphere. However, prolonged exposure showed greater modification in high-relative-humidity conditions. Dehydrogenation, dehydroxylation, chain scission, dehydroxymethylation and bond cleavage are induced during the photodegradation of cellulose chains. Nevertheless, the primary cause of cellulose photodegradation is attributed to the breakage of *β*-1-4-glycosidic bonds within cellulose chains [16]. This thorough investigation has shed light on the distinct behavior of miscanthus and flax fibers during accelerated weathering due to their differing chemical compositions. It was evident from the study that extractives play a limited role in color variation during artificial weathering for both types of fibers. Also, it should be noted that several hypotheses are possible, but the process of ageing is very complex, making it difficult to clearly identify the correct one(s). 

A consistent pattern in color variation emerged regardless of the presence or absence of extractives in miscanthus fibers. In the case of raw and extractive-free miscanthus fibers, a notable correlation was observed between the high lignin content, $∆C_{ab}^{\;*}$ and the carbonyl index after weathering. The weathering process resulted in the browning of fibers with high lignin content, which was associated with lignin photodegradation and an increase in $∆C_{ab}^{\;*}$ and the carbonyl index. This trend, however, was not observed in flax fibers (raw and EF) due to their lower lignin content and different composition compared to miscanthus. Additionally, the absence of lignin absorbance bands in the FTIR spectra of flax fibers (Supporting Information S2) suggests that the lignin in these fibers may have transformed into quinoids during retting, subsequently undergoing photodegradation. Consequently, the carbonyl index values remained stable and ${\;∆L}^{*}$ was identified as the primary factor driving the color change in flax fibers, rather than $∆C_{ab}^{\;*}$. Furthermore, it is worth highlighting that the weathering behavior of delignified flax fibers differed in terms of color variation, where the increase in $∆C_{ab}^{\;*}\;$ was responsible for the observed color changes. While various parameters and mechanisms contribute to this complex process, the initial color of the fibers emerged as an influential factor that should not be overlooked. It appears that the initial color of the fibers may influence the variations in color components during artificial weathering, adding another layer of complexity to our understanding of this phenomenon.

### 2.3. Weathering of Composites 

**Figure 8:**
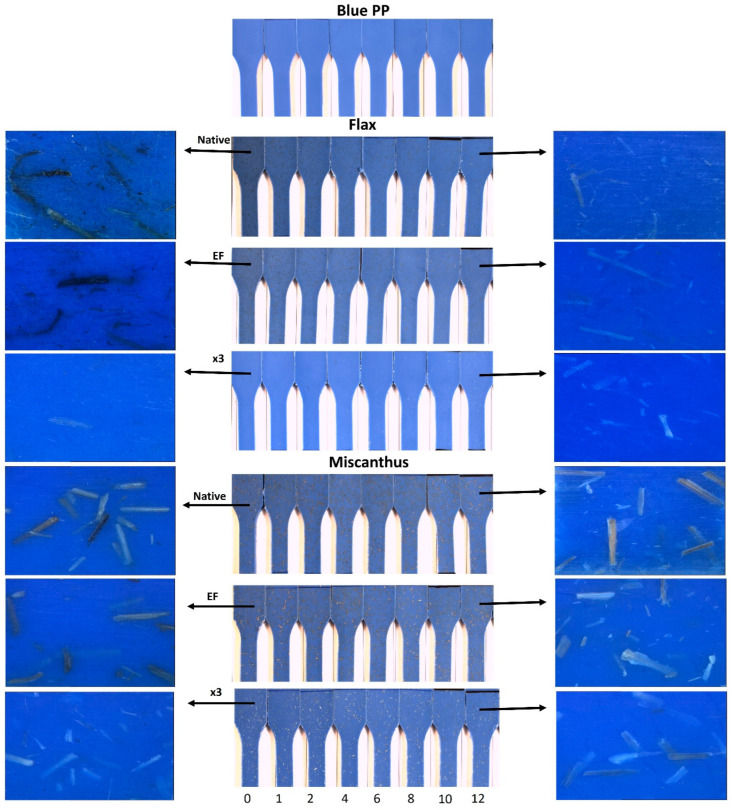
Photography of PP and PP-reinforced composites before and after several weeks of weathering.

**Figure 9:**
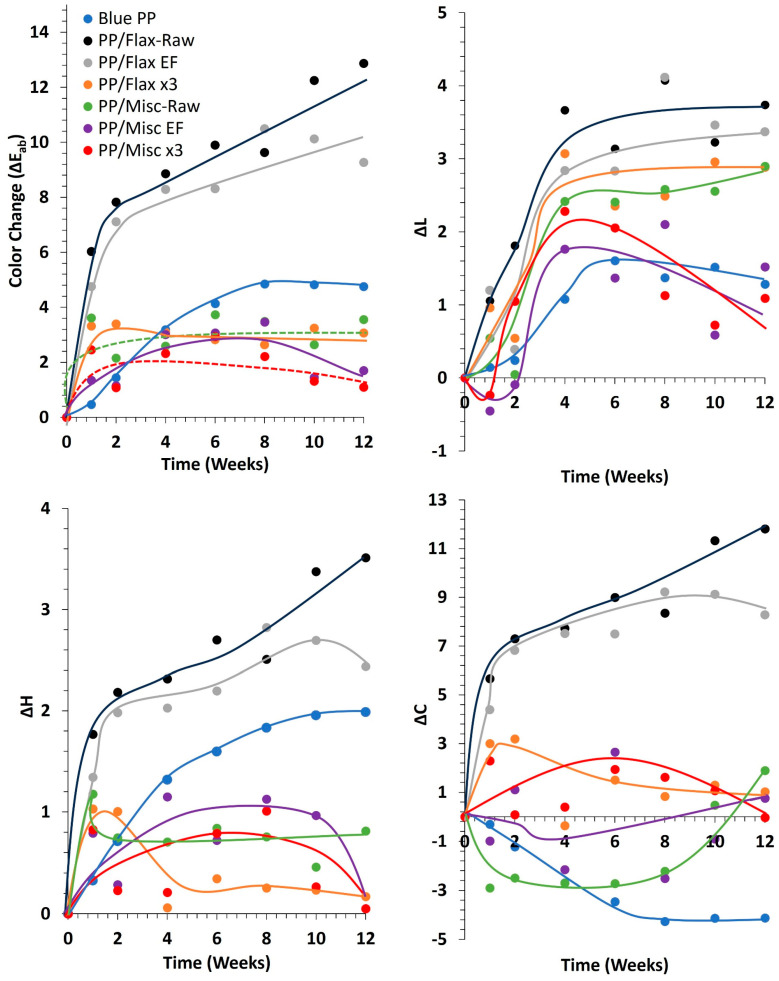
$∆E_{ab}^{*}$, Δ*C*, Δ*L* and Δ*H* variation with weathering time (weeks) for blue PP and composites (curves are added as a guideline to easily follow the results’ evolution).

A comparison of the pure PP matrix and the composites reinforced with miscanthus and flax (Raw, EF, x3) samples before and after 1, 2, 4, 6, 8, 10 and 12 weeks of weathering is depicted in Figure 8. The selection of the 12-week duration is based on the observation that total color variation tends to stabilize for most formulations, as indicated in Figure 9. The blue PP matrix appears visually stable over the 12-week period (Figure 8). Notably, upon the integration of flax and miscanthus (Raw and EF) fibers, a discernible darkening effect is observed compared to the pure PP matrix (week 0). However, this darkening, particularly characterized by the presence of dark spots, appears more pronounced in the presence of flax fibers (Raw and EF) compared to miscanthus. This darkening phenomenon for composites seems to be associated with partial fiber degradation and the formation of degradable products on the fiber surface during manufacturing. To substantiate this assumption and elucidate the influence of thermal parameters, TGA analysis was conducted on the raw fibers. 

**Figure 10:**
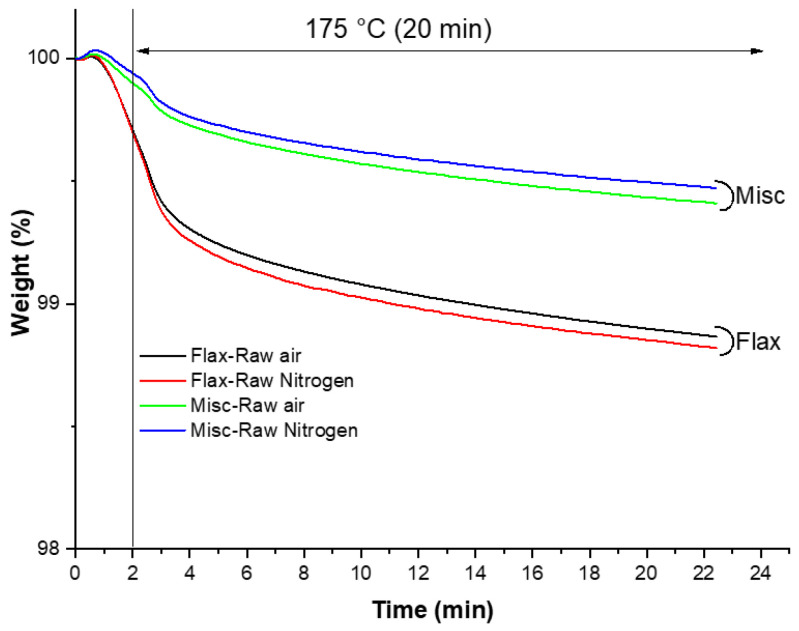
Thermogravimetric analysis (TGA) curves of Miscanthus and flax (Raw) at 175 °C in oxidative and inert atmospheres.

Figure 10 illustrates the isothermal (175 °C) thermogravimetric analysis conducted in inert and oxidative atmospheres for each anhydrous fiber. The selected isothermal temperature mirrors the thermal conditions used during the extrusion and injection processes for composite manufacturing. The results reveal accelerated thermal degradation for flax fibers, with a higher mass loss (1.2 wt%) compared to miscanthus (0.5 wt%). This difference could lead to the presence of degradation residues in flax fibers, which may contribute to the significant darkening observed, as depicted in Figure 9. Therefore, the thermal stability of fibers emerges as a critical parameter for monitoring color variation and darkening in the PP matrix prior to weathering. Understanding the thermal behavior of fibers provides valuable insights into the mechanisms underlying color changes in composite materials.

For flax fibers (Raw and EF), a notable trend of darkening disappearance and bleaching emerges with prolonged weathering, as depicted in Figure 9. Conversely, for miscanthus (Raw and EF), the darkening gradually diminishes after 12 weeks of weathering, albeit accompanied by less bleaching compared to flax. Notably, in blue PP reinforced with misc x3 and flax x3, no significant variations in color are evident for the fibers after weathering. However, it is noteworthy that, after weathering, fibers become more visible in the presence of flax x3. 

A quantitative analysis of the total color variation ($∆E_{ab}^{*}$), chroma ($∆C_{ab}^{\;*}$), lightness (${∆L}^{*}$) and hue ($∆H_{ab}^{*}$) evolution of the blue PP matrix and composites is illustrated in Figure 9. $∆E_{ab}^{*}$ in the pure blue PP experiments showed a slight increase, reaching a maximum of 4.8 after 8 weeks of weathering. Although $∆E_{ab}^{*}$ remains below the threshold for the human eye to clearly notice two different colors ($∆E_{ab}^{*}$ < 5), values below 2–3 are generally the limit for detecting a color change [60]. This quantification aligns with the qualitative observations previously presented.

The addition of miscanthus fibers (raw, EF and x3) and flax (x3) appears to induce minimal color variation in the composites compared to the matrix alone. The $∆E_{ab}^{*}$ value in the presence of misc-Raw is 3.5 after 12 weeks of weathering. Lower color variation is observed in Misc EF composites, where the $∆E_{ab}^{*}\;$ value reached 2 after 12 weeks of weathering. It is noteworthy that color change could be attributed to the loss of methoxyl groups and the formation of paraquinone chromophoric structures after lignin oxidation, which are reduced to hydroquinones and cause a photobleaching phenomenon [22,58,65]. Better results in terms of color variation are observed in Misc-x3, where the $∆E_{ab}^{*}\;$ value reached 1.1 after 12 weeks of aging, highlighting better color stability. Only experienced observers can notice the difference [60]. 

This tendency aligns with the findings of Peng et al. (2014) [28], where the impact of UV-accelerated weathering on the surface properties of PP composites reinforced with wood flour, lignin and cellulose was explored. Their results indicated an acceleration of discoloration or photobleaching that appeared to be correlated with lignin content. However, the stabilization effect and antioxidant properties of lignin act as a shield against photodegradation in the composites, as corroborated by the mechanical outcomes. Additionally, better color stability was observed in the presence of cellulose despite the formation of superficial microcracks, the reduction in flexural properties and the increase in the surface’s hydrophilic character. Flax x3 composites also demonstrated good results in terms of color variation ($∆E_{ab}^{*}$ = 3), remaining higher than Misc x3. This disparity could be elucidated by qualitative observations (Figure 8), where Flax x3 fibers appear more visible after weathering, potentially inducing greater color variation.

However, it is noteworthy that the color variation, $∆E_{ab}^{*}$, is significant for flax (raw and EF) composites, reaching values of 12.8 and 10 after 12 weeks of weathering. This denotes a noticeable difference in color that is easily discernible by the human eye [60]. Consequently, flax (raw and EF) composites demonstrate lower color stability compared to the blue PP matrix and the other fiber-reinforced composites. 

For a better understanding of the evolution of $∆E_{ab}^{*}$, comparisons are made among $∆H_{ab}^{*}$, ${∆L}^{*}$ and $∆C_{ab}^{\;*}$ for the different formulations in Figure 9. In the case of blue PP, the slight increase in $∆E_{ab}^{*}$ is associated with the combined modifications of $∆H_{ab}^{*}$ and ${∆L}^{*}$ increasing from 0 to 1.9 and from 0 to 1.6, respectively, and a decrease in $∆C_{ab}^{\;*}$ (from 0 to −4). $∆H_{ab}^{*}$ for the composites containing miscanthus (Raw, EF and x3) and flax (x3) remains lower than that of the blue PP (value < 1). However, in the presence of flax (raw and EF), $∆H_{ab}^{*}\;$ increases progressively and significantly over time with weathering (value > 2–3). This evolution underscores a notable change in hue for composites containing flax (raw and EF) compared to the other formulations.

For composites with miscanthus (raw, EF and x3) and flax (x3), the trend in $∆E_{ab}^{*}$ appears to be associated with minimal variation in $∆C_{ab}^{\;*}$ values, ranging between −3 and +3, and an increase in ${∆L}^{*}$ between 1 and 3. However, for composites reinforced with flax (raw and EF), the higher $∆E_{ab}^{*}\;$ obtained is primarily attributed to a significant increase in $∆C_{ab}^{\;*}$ (8–11), and to a lesser extent, to slight increases in ${∆L}^{*}$ (3.3–3.7) and, as already said, $∆H_{ab}^{*}$ (2.4–3.5). This shift in color-change trends in the composites was unexpected, considering that for both miscanthus and flax fibers alone (raw and EF), $∆E_{ab}^{*}$ changes significantly, and that for flax (raw and EF) alone, ${∆L}^{*}\;$ was mainly responsible for $∆E_{ab}^{*}\;$ variation, not $∆C_{ab}^{\;*}$. This suggests that the color-change tendency of fibers alone cannot be used to predict the color change of a blue-colored matrix. The findings regarding color change in the composites of this study align with those of Badji and coworkers [23], who explored the outdoor and under-glass natural weathering of uncolored pure PP reinforced with hemp fibers. In addition to their mechanical, chemical and microstructural analyses, the authors conducted colorimetric measurements based on the CIE L*a*b* system to track changes in chromaticity and lightness. The authors concluded that hemp/PP composites were generally more sensitive to weathering than neat PP, regardless of the weathering conditions, due to a significant impact on lightness evolution and a yellowing effect caused by the decomposition of lignin structure and the thermal oxidation of hemicelluloses. They attributed higher yellowing to the higher temperatures reached during under-glass aging. Therefore, despite the addition of blue coloration in our study, the overall color variation resulting from the presence of flax (raw and EF) follows a similar trend to that observed in the literature for hemp composites.

Furthermore, a recent study by Nasri and coworkers [24] evaluated the influence of UV weathering on the mechanical properties and drop-weight impact performance of polypropylene biocomposites reinforced with short flax and pine fibers. Pine fibers have significantly higher lignin content, but their length is half that of flax fibers. As a result, flexural strength and impact resistance increase in the presence of flax, while UV aging is mitigated in the presence of pine fibers. In their study, the authors monitored the discoloration phenomenon based on the CIE L*a*b* system. The color difference was quantitatively assessed by the values of Δ*E*, which showed a significant evolution up to 480 h of exposure, followed by a moderate variation until 960 h. This evolution differed depending on the type of fiber present and was more pronounced for flax, correlating with changes in the luminosity parameter and lignin photodegradation (as lignin structures differ between the two fibers) and the formation of different types of quinone structures. The authors linked the color change to lignin structure, with the G unit representing more than 90% of pine fibers, while flax fibers contain 72% G unit, 13% H unit and 15% S unit (p-hydroxyphenyl (H), vanillin (G), syringaldehyde (S)). In the present study, miscanthus lignin [51] appears to have lower G (52%) and H (4%) units but higher S units (44%) compared to flax fibers (72% G, 13% H, 15% S) and flax shives (87% G, 5% H, 8% S) [63]. Despite the higher content of G units in flax, the color variation was more pronounced for this fiber. Hence, another parameter that may be involved in this scenario may be the thermal stability of the fibers, as demonstrated previously (see Figure 10), which appears to impact the fibers’ colors during the shaping steps (processing). Consequently, color variation is more significant in the presence of fibers with lower thermal stability due to partial degradation during processing. It should be concluded that no correlation appears between the observations made on the fibers and those on the composites.

## 3. Materials and Methods

### 3.1. Materials 

Flax and miscanthus fibers sourced from Normandy, France, were supplied by Addiplast company (Saint-Pal-de-Mons, France). Miscanthus underwent a sieving process to remove any dusty components. Subsequently, fibers that passed through a 400 µm mesh but that were retained by a 250 µm mesh were utilized. Flax fibers were used as received, without any prior treatment. The materials used in the study included sodium chlorite (80% purity) from Honeywell (Saint Germain en Laye, France), glacial acetic acid (99.7% purity), sodium chloride (99.5% purity), ethanol (96% purity) from PANREAC (Castellar del Vallès, Spain) and toluene (99.5% purity) from Merck KGaA (Darmstadt, Germany), all used in their as-received state. The blue stabilized polypropylene matrix was provided by Plastic Omnium (Lyon, France).

### 3.2. Methods

#### 3.2.1. Elemental Nitrogen, Sulfur, Carbon and Hydrogen Contents

The elemental nitrogen, sulfur, carbon and hydrogen contents of native flax and miscanthus fibers were analyzed using a Flash EA 1112 (Thermo Finnigan, San Jose, CA, USA). In this process, 1.5 mg of fiber undergoes thermal degradation at 1000 °C for 15 s in the presence of tungstic anhydride within an oxidizing atmosphere. Subsequently, the combustion gases (CO_2_, H_2_O, SO_2_, NO_x_) are subjected to gas chromatography analysis. It is important to note that any NO_x_ formed is reduced in the presence of copper, ultimately forming N_2_. The “Eager 300” software (version 2.3) is then employed to calculate the mass percentage of the different elements present in the fibers.

#### 3.2.2. Ash Content

The ash content of various fibers was determined following ASTM D 1102–84 [66] guidelines using a Nabertherm LHT muffle furnace (Lilienthal, Germany). Initially, empty alumina crucibles undergo calcination at 600 °C for 3 h, followed by cleaning with ethanol, drying at 105 °C overnight, cooling in a desiccator and subsequent weighing. Subsequently, 2 g of dry-weight fiber is placed into a crucible. Employing a heating rate of 20 °C/min, the fibers are thermally degraded at 600 °C for 6 h in ambient air. Following this, the crucibles, along with their residual inorganic matter content, are deposited and cooled in a desiccator prior to weighing. The reproducibility of the analysis results is ensured by conducting 2 tests on each fiber. The percentage of ash is then calculated based on the initial dry mass of the fibers and that of the residues using Equation (1).

(1) $$Ash,\%=\frac{W_{1}}{W_{2}}\times 100$$

where W_1_ is mass of ash and W_2_ is mass of anhydrous fibers.

#### 3.2.3. Determination of Soluble Extractive Content

The determination of soluble extractive content in the fibers, primarily comprising waxes, fats, resins and certain gums, adhered to ASTM D 1107–96 standards [67]. Initially, a dry mass of 2 g of fiber is placed in a cellulose cartridge, which is then sealed with fiberglass cotton to prevent fiber loss during extraction. The entire setup undergoes extraction using a Soxhlet extraction device for 6 h, with the solvent (ethanol/toluene 2/1–300 mL) maintained above its boiling point throughout. This enables 12 siphons per hour, totaling 72 siphons per extraction. The solvent containing the extractives is collected in the extraction flask and subsequently evaporated using a rotary evaporator. Following evaporation, the flask containing the extractives without solvent undergoes drying in an oven at 105 °C overnight. Once cooled in a desiccator, it is weighed. The extractive contents are determined based on an average of six tests, as outlined in Equation (2).

(2) $$Extractives,\%=\frac{W_{e}}{W_{i}}\times 100$$

where W_e_ is the mass of extractives in g and W_i_ is the mass of dry fibers in g.

#### 3.2.4. Determination of Holocellulose Content

The determination of holocellulose content followed the method outlined by Moussa and coworkers [68]. Initially, 2 g of fiber without extractives is combined with 167 mL of deionized water in a 250 mL flask (83.3 mL/g of fibers). The mixture is heated in the presence of a refrigerant until 70 °C is reached. Sodium chlorite (NaClO_2_—0.67 g/g of fibers) and glacial acetic acid (CH_3_COOH—0.67 mL/g of fibers) are then added to the mixture, which is brought to reflux under continuous stirring for 2 h. This process involves the addition of NaClO_2_ and CH_3_COOH three times, with a maximum heating time of 6 h. The result is a whitish solid residue, primarily composed of holocellulose, due to oxidative delignification. The residue is vacuum-filtered using a No. 2 sintered glass previously dried and weighed under vacuum. It is then washed several times with deionized water until neutralization with ethanol. The sintered glass containing the holocellulose is dried in an oven at 60 °C overnight, cooled in a desiccator and finally weighed. The holocellulose content is determined based on an average of 3 tests, as per Equation (3).

(3) $$Holocellulose,\%=\frac{W_{f}}{W_{i}}\times 100\;w$$

where W_f_ is the mass of holocellulose in g and W_i_ is the mass of dry fibers in g. The calculated final percentage is normalized considering the initial extractive contents.

#### 3.2.5. Determination of Cellulose Content 

The determination of cellulose content adhered to the method outlined by Moussa and coworkers [68]. Initially, in a 250 mL flask, a dry mass of 1.5–2 g of fiber, without extractives and delignified (holocellulose), is combined with 10 mL of a sodium hydroxide solution (17% m/m). After 5 min of soaking and crushing the fibers with a ground glass rod, an additional 5 mL of the NaOH solution is added. The mixture is carefully stirred with the glass rod. Following another 5 min, 5 mL of the NaOH solution is added and stirred and the process is repeated until a total treatment time of 45 min is reached. Subsequently, 33 mL of distilled water is added to the mixture, which is then allowed to stand at room temperature for 1 h. The mixture is then filtered on a Buchner using a No. 2 sintered glass of known initial weight. The fibers are washed carefully with 100 mL of NaOH 8.3% (m/m), followed by abundant washing with deionized water. Next, the fibers undergo washing for 3 min with 15 mL of acetic acid (10% m/m). A final washing step entails rinsing with 250 mL of additional deionized water and 10 mL of ethanol. The crucible containing the cellulose is dried in an oven at 105 °C overnight, then cooled in a desiccator containing silica gel for 30 min before being weighed. The percentage of alpha-cellulose is calculated based on an average of 3 tests, as per Equation (4).

(4) $$Alpha-cellulose,\%=\frac{W_{2}}{W_{1}}\times 100$$

where W_2_ is the mass of the oven-dried alpha cellulose residue and W_1_ is the dry mass of the starting holocellulose fibers. Note that the overall cellulose content was recalculated by normalization with respect to the native fiber considering the initial lignin and extractives.

#### 3.2.6. Determination of Klason Lignin Content

The Klason lignin content was determined on extractive-free fibers following the laboratory analytical procedure (LAP) outlined by the National Renewable Energy Laboratory [69]. Initially, 175 mg of dry biomass is combined with 1.5 mL of 72% sulfuric acid in a 50 mL plastic centrifugation tube. The mixture is stirred for 1 h in a water bath at 30 °C for hydrolysis. Following hydrolysis, 42 mL of ultrapure water is added to dilute the sulfuric acid concentration to 4% (m/m). The tube containing the mixture is then heated to 120 °C under a pressure of 1.5 bar in an autoclave for 1 h. Klason lignin is subsequently recovered from the cooled solution after Buchner filtration using a ceramic “Gooch” crucible containing a glass fiber filter with a porosity of 1.2 µm. The mass of lignin is measured after washing and drying in an oven at 105 °C overnight and cooling in a desiccator. The normalized Klason lignin content and standard deviation are then calculated from three independent experiments performed under the same conditions according to Equation (5).

(5) $$Klason\;Lignin,\%=\frac{W_{L}}{W_{i}}\times 100$$

where W_L_ is the mass of lignin residue recovered and W_i_ is the initial mass of fibers with extractives.

#### 3.2.7. Fiber and Disc Preparation 

Native flax (Flax-Raw) and miscanthus fibers (Misc-Raw) underwent distinct treatments aimed at modifying their chemical composition, conducted on a large scale. Extractive-free flax (Flax EF) and miscanthus (Misc EF) fibers were prepared by following a previously outlined protocol, wherein 30g of fiber was utilized for each extraction. Upon completion of the extraction process, the fibers were thoroughly dried in a fume hood for 24 h before further use. Flax and miscanthus fibers underwent delignification treatments following the same protocol. For several repetitive experiments, 60 g of fibers underwent three (Flax x3 and Misc x3) treatments with sodium chlorite (0.67 g/g of fibers) and acetic acid (0.67 mL/g of fibers) at 70–75 °C under mechanical stirring at 600 rpm. Subsequently, the fibers were filtered on a Buchner using a polyamide fabric with a porosity of 10 µm (Buisine, Clermont de l‘Oise, France; ref. PA-03-10-2). The fibers were then washed with water until neutralization, followed by a final washing with ethanol before drying at room temperature in a fume hood for 24 h. For the preparation of compressed discs, amounts of 500 mg of raw, extractive-free and delignified flax and miscanthus fibers were compressed for 2 min at 105 °C and 30 kN using a “Prontopress-2, Struers A/S, Copenhagen, Denmark” press to form discs with a diameter of 2.5 cm and thickness of 2 mm. The discs were recovered after cooling to room temperature for 4 min.

#### 3.2.8. Composite Preparation

The native and modified miscanthus and flax fibers underwent drying in an oven at 60 °C overnight before compounding. PP-based pellets were conditioned in a compressed air dryer (Piovan, DS503, Nurieux-Volognat, France) at 60 °C. The PP and the composites were extruded at 175 °C using a twin-screw extruder (BC21 Clextral 900 mm, Firminy, France). Fibers were manually dosed and mixed with the melted PP matrix to achieve a specific ratio of 5 wt%. The rotor screw speed was 196 rpm, the total flow rate of the materials was 6 kg·h^−1^, and 1.5 kg of material was produced per composition. The extruded strand was cooled in a water bath and subsequently granulated. The resulting PP and PP composite granulates were dried in the compressed air dryer at 60 °C for 24 h. Samples with a thickness of 4 mm were shaped into ISO-1A dumbbells using an injection molding machine (KraussMaffei, KM50-180CX, Parsdorf, Germany) at 185 °C.

#### 3.2.9. Accelerated UV Weathering 

Nine discs of each fiber composition and seven PP-based formulations in the form of ISO-1A dumbbells cut in half were placed in a QUV/se accelerated weathering tester (Q-Lab, Cleveland, OH, USA). Aging was conducted under conditions partially conforming to ISO 4892-3 [70] (without water spraying) for a total of 20 weeks (3360 h) for discs and 12 weeks for composites. Samples underwent repetitive 12 h cycles comprising 8 h of UV exposure at 340 nm wavelength, with an irradiance of 0.76 W/m^2^ and a temperature of 60 °C, followed by 4 h of dark exposure at 40 °C. The samples were extracted at various stages of ageing for evaluation and analysis, then compared to the native samples.

#### 3.2.10. Spectrophotometer Analysis

For comparative purposes, color measurements were monitored with a polychromator-type spectroradiometer (Konica-Minolta CS-2000, Tokyo, Japan), which allows light measurements in order to obtain the spectral reflectance of color surfaces. Even though measured surfaces are non-uniform, the light entering the system from the measurement area is mixed and considered virtually uniform. So, our goal was to maximize the measured area in order to average the color of the samples. The aperture (measuring angle) was fixed at 1° and each sample was placed and analyzed at 86 cm from the apparatus bottom edge to maximize the measured area. Two light sources (continuous spectrum with spectral characteristics close to D50 standard illuminant (designed to mimic the spectral characteristics of natural daylight at noon)) were set at 45° from the measured surfaces, and the spectroradiometer was set vertical to the surface. To obtain the spectral reflectance of the color surfaces, spectral radiance was measured on the sample’s surface and on a white calibration plate in the same position. Using the average of three measurements for each area, the CIE L*a*b* 1976 values were calculated, along with chroma (C*), which measures the intensity of a color, and hue angle (h*), which is the attribute of a color that determines its position in the color wheel. The color difference ($∆E_{ab}^{*}=\sqrt{{∆L}^{2}+{∆a}^{2}+{∆b}^{2}})$ between the different samples, based on differences in lightness (${∆L}^{*}$), chroma ($∆C_{ab}^{*}$) and hue ($∆H_{ab}^{*}$), was then calculated and compared. ∆*a* and ∆*b* are the differences in the a* and b* components between two colors, measuring the perceived changes between green and red hues and blue and yellow hues, respectively. ${∆L}^{*}$ increases and decreases correspond, respectively, to color lightening and darkening. $∆C_{ab}^{*}$ represents the difference in chroma: an increase in *C* corresponds to an increase in saturation. $∆H_{ab}^{*}$ corresponds to the difference in hue angles. Note that, for each series, the non-weathered sample is used as the reference in our comparison process.

#### 3.2.11. Scanning Electron Microscope (SEM) Observation 

The surface morphology of the fiber discs and composites was assessed both in their native state and after 20 weeks of weathering using an environmental scanning electron microscope (FEI Quanta 200 FEG, Hillsboro, OR, USA). Each sample was deposited onto the sample holder and observation was conducted. High-resolution backscattered electron micrographs were captured under high vacuum conditions at 10 mm with a voltage of 12.5 kV, with a magnification of 50× for the fiber discs and 200× for the PP dumbbells.

#### 3.2.12. Attenuated Total Reflectance-Fourier-Transform Infrared Spectroscopy (ATR-FTIR)

The surfaces of the fiber discs underwent analysis using a Vertex 70 single-reflection diamond ATR-FTIR (Bruker Corporation, Ettlingen, Germany). Baseline-subtracted spectra for each sample collected at various exposure times were recorded with 32 scans and a resolution of 4 cm^−1^. To facilitate comparison, baseline correction and normalization to 1 absorbance unit for the highest band at 1030 cm^−1^ (Temiz et al., 2007 [57]) were conducted for each spectrum. To monitor changes in carbonyl content, the ratio of carbonyl groups (I_1730_/I_1370_) was calculated by dividing to the relative intensity of the absorption bands at 1730 cm^−1^ (associated with the C=O stretching vibration of carbonyl groups) by the intensity of the absorption peak at 1370 cm^−1^ (associated with C-H bending vibrations) [47].

#### 3.2.13. Thermogravimetric Analysis (TGA)

The thermal stability of the fibers was assessed using a PerkinElmer TGA 800 thermogravimetric analyzer (Shelton, CT, USA) to mimic the effects of temperature encountered during the composite preparation processes. Approximately 9–10 mg of each sample was subjected to the following heating program at a rate of 10 °C·min^−1^ under either a nitrogen or air atmosphere (40 mL·min^−1^):

1. Heating from room temperature to 105 °C;
2. Isothermal heating at 105 °C for 10 min to remove residual moisture;
3. Fast heating from 105 °C to 175 °C for 2 min;
4. Isothermal heating at 175 °C for 20 min.

The thermograms obtained from steps 3 and 4 were recorded and the weight loss as a function of time was analyzed and compared to evaluate the thermal stability of the fibers.

## 4. Conclusions

This study investigated the impact of flax and miscanthus fiber chemical compositions on the weathering of the fibers themselves and of extruded–injected PP composites containing 5 wt% of the fibers. The following conclusions were drawn:

1. The weathering of fibers was found to be influenced not only by the presence of lignin, but also by their type, composition and initial color. Lignin presence and content in miscanthus significantly influenced color variation, while extractive presence showed a limiting effect on color variation for both fibers.
2. Both raw and EF flax and miscanthus exhibited color changes, reaching the same value for total color variation ($∆E_{ab}^{*}$) of 12–13 after 20 weeks of weathering. $∆E_{ab}^{*}$ variation is linked to $∆C_{ab}^{\;*}$ for miscanthus and to ${∆L}^{*}\;$ for flax.
3. Miscanthus raw and EF fibers darkened over time, whereas Flax-Raw and EF fibers lightened. This behavior correlated with higher lignin content and lignin type in miscanthus, along with significant differences in carbonyl (C=O) formation and $∆C_{ab}^{\;*}$ increase.
4. Delignified fibers, particularly flax x3, demonstrated better color stability.
5. The preparation process of composites significantly influenced fiber color before weathering, thereby affecting the color evolution during weathering. Composites demonstrated better color stability when incorporating miscanthus (Raw, EF and x3) and delignified flax fibers. This stability was attributed not to the type of lignin present but to the thermal stability of the fibers, as suggested by the TGA results. The thermal stability of the fibers during processing plays a crucial role in color variation by producing darker degradable products. However, no direct correlation was found between the weathering of fibers alone and their behavior after being integrated into the PP blue matrix. The color variation trends of standalone fibers differed from those observed after their integration into the matrix, indicating that the extrusion and injection processes contributed to pre-weathering color changes likely due to the thermal modification of the fibers.
6. Another hypothesis that could explain this behavior involves the chemical interactions between the PP matrix and the fibers, possibly due to the disruption of the PP stabilizer by the presence of the fibers. Additionally, the color variation might be attributed to an interference phenomenon related to the blue color of the matrix. Further investigation into these hypotheses is recommended to fully understand the underlying mechanisms.
7. By analyzing the results of this study, we aim to develop strategies to enhance fiber stability during composite preparation and aging. Efforts are underway to improve the stability of these fibers.

## Figures and Tables

**Table 1:** Elemental analysis results for native flax (Flax-Raw) and miscanthus (Misc-Raw).

	Element, Content (%)	
Fibers	C	H
Flax-Raw	42.1	6.2
Misc-Raw	46.0	6.0

**Table 2:** Klason lignin content for the native and treated miscanthus and flax fibers.

Fibers	Klason Lignin (%)
Misc-Raw	23.00 ± 0.54
Misc EF	23.52 ± 0.55
Misc x3	2.50 ± 0.40
Flax-Raw	9.10 ± 0.58
Flax EF	9.50 ± 0.61
Flax x3	0.82 ± 0.23

## Data Availability

Data are contained within the article and Supplementary Materials.

## Supplementary Materials

The following supporting information can be downloaded at: https://www.mdpi.com/article/10.3390/molecules29163945/s1, Supporting Information S1: FTIR signals and corresponding assignment for *Miscanthus x giganteus* particles in this study; Supporting information S2: IR spectra of flax (raw, EF and x3) before weathering; Supporting Information S3: IR spectra of flax (raw) before and after 1 and 20 weeks of weathering); Supporting Information S4: IR spectra of flax (x3) before and after 1 and 20 weeks of weathering; Supporting Information S5: IR spectra of miscanthus (x3) before and after 1 and 20 weeks of weathering.
