# Supplementary material for: Enhancing Insight into Photochemical Weathering of Flax and Miscanthus: Exploring Diverse Chemical Compositions and Composite Materials
Source: Molecules. 2024 Aug 21;29(16):3945. doi: 10.3390/molecules29163945 (PMC11357340; doi:10.3390/molecules29163945)

Supporting information S1:

FTIR signals and corresponding assignment for miscanthus x giganteus particles in this study [43, 48, 49, 51, 52, 56]

| Wave number ($cm^{-1}$) | Functional group | Assignment | Moieties |
|---|---|---|---|
| 3324 | O-H | Stretching | Cellulose, hemicellulose, lignin, and extractives |
| 2917 | $CH_2$ | Asymmetric stretching | Extractives |
| 2850 | $CH_3$ | Symmetrical vibration | Extractives |
| 2895 | C-H or $CH_2$ | Stretching | Cellulose, hemicellulose, and lignin |
| 1730 | C=O | Stretching | Mainly for Hemicellulose (weakly for lignin and extractives) |
| 1640 | C=O | Stretching | Lignin (weakly) |
| | H-O-H | Deformation | Absorbed water in cellulose and hemicellulose |
| 1600 and 1510 | C=C | Stretching | Mainly for lignin and |
| 1456 | C-H | Deformation | weakly for extractives |
| 1421 and 1370 | H-C-H / O-C-H | Bending vibration | Cellulose |
| | C-H (in $CH_3$ and phenol) | Deformation | Lignin and extractives |
| 1429 | H-C-H | Bending vibration | Cellulose |
| 1317 | $CH_2$ | Rocking vibration | Mainly cellulose |
| | C-O | Stretching | Lignin (Weakly) |
| 1240 | C-O | Stretching | Lignin, cellulose, and Hemicellulose |
| 1159 | C-O-C | Asymmetric stretching | Cellulose and hemicellulose |
| | C-H | Stretching | Extractives and lignin |
| 1103, 1029, 985 | C-C, C-OH, C-H | Vibration | Cellulose and hemicellulose |
| 833 | C-H | Out-of-plan deformation | Lignin |
| 663 | C-OH | Out-of-plan bending | Cellulose |
| 558 | | | |

Supporting information S2: IR Spectra of flax (raw, EF and x3) before weathering

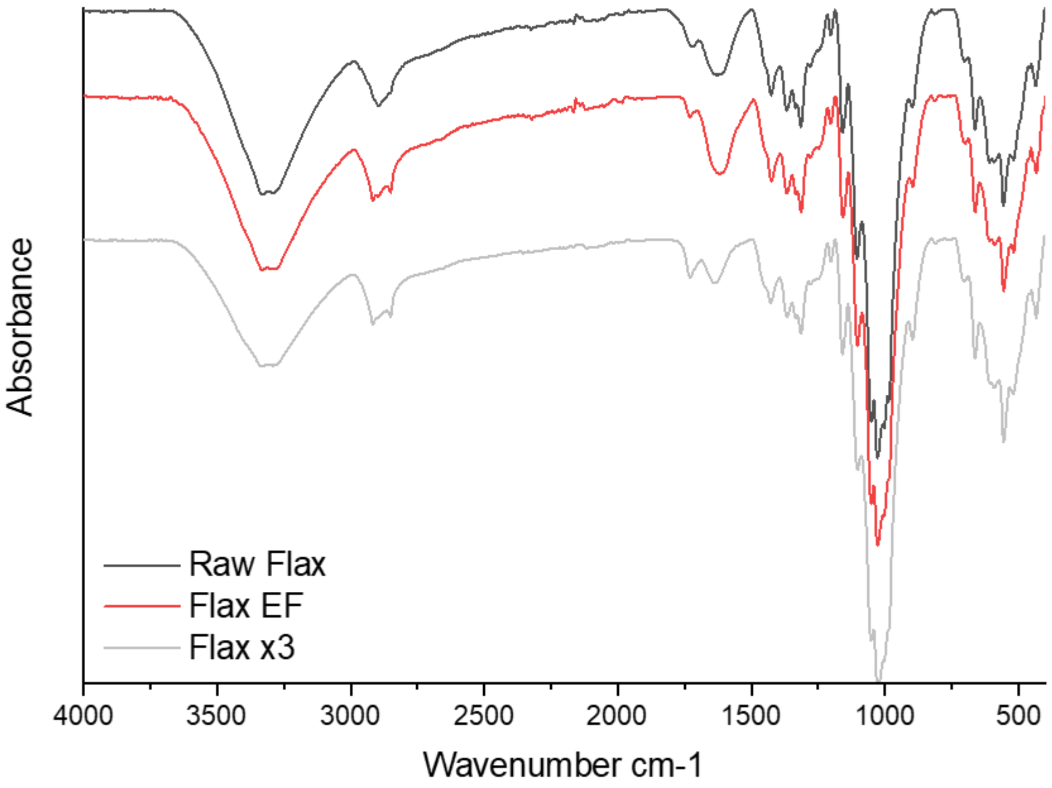


Supporting information S3: IR Spectra of flax (raw) before and after 1 and 20 weeks of weathering)

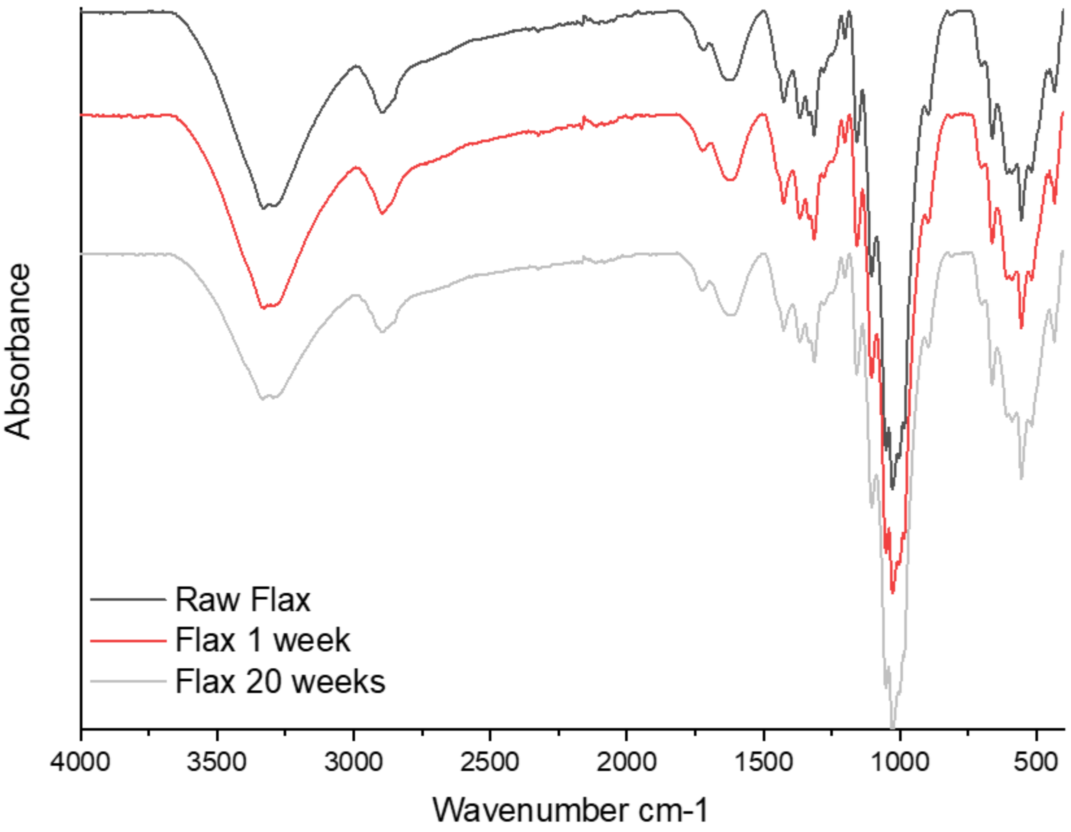

Supporting information S4: IR Spectra of flax (x3) before and after 1 and 20 weeks of weathering)

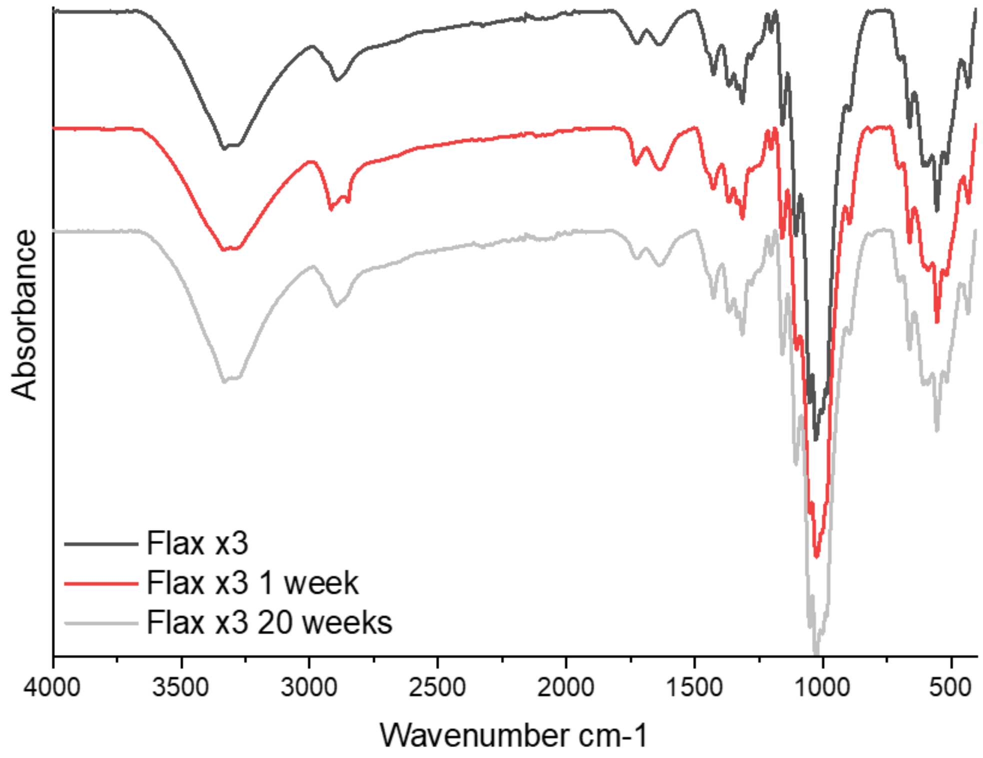


Supporting information S5: IR Spectra of miscanthus (x3) before and after 1 and 20 weeks of weathering)

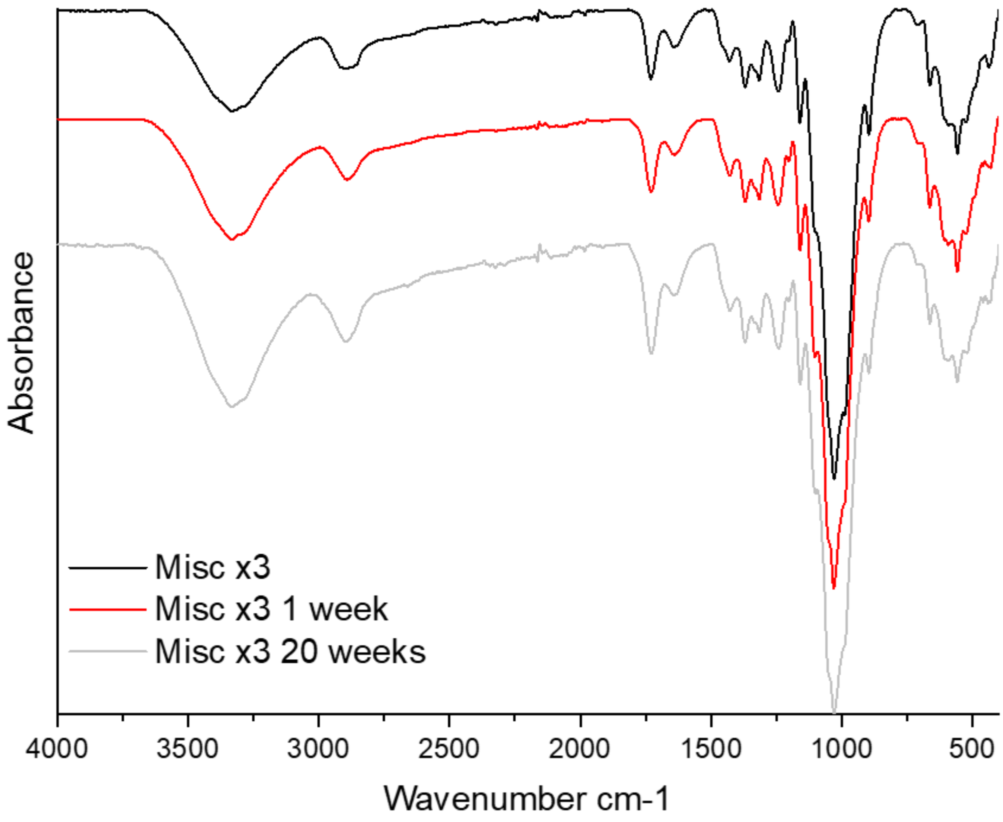

Supplement: Supplementary file 1 [file molecules-29-03945-s001.zip › molecules-3126747-supplementary.pdf]
